# Comparison of the Effect of Two Purification Methods on the Immunogenicity of Recombinant Outer Membrane Protein H of* Pasteurella multocida* Serovar A:1

**DOI:** 10.1155/2016/2579345

**Published:** 2016-01-17

**Authors:** Arunee Thanasarasakulpong, Pichayanut Poolperm, Weerapongse Tangjitjaroen, Thanya Varinrak, Takuo Sawada, Dirk Pfeiffer, Nattawooti Sthitmatee

**Affiliations:** ^1^Faculty of Veterinary Medicine, Chiang Mai University, Chiang Mai 50100, Thailand; ^2^Laboratory of Veterinary Microbiology, Nippon Veterinary and Life Science University, Tokyo 180-8602, Japan; ^3^Veterinary Epidemiology, Economics and Public Health Group, Royal Veterinary College, London AL9 7TA, UK

## Abstract

Recombinant outer membrane protein H (rOmpH) of* Pasteurella multocida* strain X-73 can be purified using affinity chromatography but this adversely affects its immunogenicity. The current study presents the results from an intervention study comparing the immunogenicity of rOmpH purified using electroelution with rOmpH purified using affinity chromatography and native OmpH purified using electroelution and a nonimmunized control group. Chickens immunized with rOmpH purified using electroelution produced the highest ELISA antibody levels against* P. multocida* strains. Chickens in each of the 5 treatment groups were split into two subgroups for challenge with two different* P. multocida* strains. The average number of adhesions to CEF cells was statistically significantly lower in sera from chickens immunized with rOmpH or native OmpH purified using electroelution than in those of the three other treatment groups. The survival amongst chickens immunized with rOmpH or native OmpH purified using electroelution indicated high levels of protection. In contrast, survival probability was zero or low in the groups immunized with rOmpH purified using affinity chromatography and in the nonimmunized group. These findings show that the rOmpH purified using electroelution retains its immunogenicity and stimulates high levels of protection in chickens against* P. multocida* infection.

## 1. Introduction

Recombinant proteins with 6×His-tagged protein are routinely purified by a Ni-NTA affinity chromatography as recommended by their manufacturers. However, Luo et al. [1] and Rimler [2] suggested that this process resulted in a change in the structure of the recombinant outer membrane protein H (rOmpH) of avian* Pasteurella multocida* strain X-73 affecting its immunogenicity. The OmpH, a porin protein, is stable in the homotrimer form at room temperature and was fully dissociated into monomers which correlated with the unfolded or denatured form of protein after purification of the protein using a denatured condition of affinity chromatography. In contrast, Sthitmatee et al. [3] successfully improved the immunogenicity of rOmpH using a hybrid condition of affinity chromatography to purify rOmpH. But this method is unstable, is of low reproducibility, and results in a high loss of protein yield [3]. This suggests that affinity chromatography may be unsuitable for purification of this recombinant protein. The electroelution method is widely used for analytic purposes [4–6]. The method employs polyacrylamide gel electrophoresis (PAGE) which is easy to perform and has high resolution and good reproducibility. This system also has advantages in terms of having high loading capacity of sample protein and allowing easy monitoring of the elution process. Previous studies using electroelution to purify target proteins showed that the method provides an effective purification method for protection of immunogenicity of the target proteins [7–10]. Interestingly, the method has been used for purification of a native form of OmpH while completely protecting its immunogenicity [8]. This suggests that the electroelution method could also be applied for purification of rOmpH. The present intervention study aimed at comparing the performance of the electroelution and affinity chromatography methods for purification of the rOmpH in terms of their effect on the immunogenicity of the recombinant protein.

## 2. Materials and Methods

### 2.1. Experimental Design

An intervention study was used with five treatment groups; there were four immunized groups and one nonimmunized group (Table 1). The treatments included group 1, immunized with rOmpH purified using electroelution, group 2, immunized with rOmpH purified using a denatured condition of affinity chromatography [1], group 3, immunized with native OmpH purified using electroelution [8], group 4, immunized with incomplete Freund's adjuvant, and group 5, a nonimmunized group, respectively. Each of the five treatment groups was divided into two subgroups, one challenged with* P. multocida* serovar A:1 and the other with serovar A:3. There were therefore in total 10 treatment-challenge subgroups. Hisex brown chickens at the age of 21 weeks sourced from RPM Farm & Feed Co. Ltd., Chiang Mai, Thailand, were used in this study. The outcome variables which were compared between the treatment groups were immunological and clinical parameters. The former consisted of serum antibody and cell adhesion levels in response to infection challenge, both measured after vaccination, and the latter of survival of chickens. The chickens were randomly allocated to the 10 treatment-challenge subgroups, with 10 chickens in each of the six subgroups immunized with rOmpH or OmpH purified using electroelution and 5 chickens in each of the four other subgroups. The group size of 10 was sufficient to detect a reduction in mortality in a pairwise comparison from 100 to 40% at 95% confidence level and with 80% power. A group size of 10 in each of the OmpH or rOmpH immunized and 5 in each of the two comparison groups allowed for detection of a reduction in mortality from 100 to 30% at 95% confidence level and with 80% statistical power. Chickens in groups 1–3 were intramuscularly immunized two times at a 2-week interval with a total volume of 1 mL of 100 *μ*g rOmpH or OmpH emulsified with an equal volume of incomplete Freund's adjuvant (Sigma-Aldrich), specifically group 1 with rOmpH purified using electroelution, group 2 with a denatured condition of affinity chromatography, and group 3 with native OmpH purified using electroelution. Chickens in group 4 were intramuscularly immunized with incomplete Freund's adjuvant in PBS buffer and group 5 was not immunized (Table 1). Chickens in the 4 immunized groups were immunized twice, once on day 0 and again on day 14. All groups were intramuscularly challenged with either approximately 2 × 10^6^ cfu/mL or 4.3 × 10^6^ cfu/mL of live* P. multocida* strains X-73 or P-1059 [3], respectively, at 2 weeks after the second immunization.

Blood samples from each chicken were collected from the wing veins on days 0, 7, 14, 21, and 28 after immunization. Sera were assayed using western blot, indirect ELISA, and the adhesion inhibition assay. All chickens were observed every day for clinical signs and behavioral changes during the experiments. Once chickens showed clinical signs of the disease, they were euthanised according to the protocol in the AVMA guideline for the euthanasia of animals, version 2013 [11]. The experimental use of animals in this study was approved by the animal welfare and laboratory animal ethics committee of the Faculty of Veterinary Medicine, Chiang Mai University, Chiang Mai, Thailand (approval number R15/2555).

### 2.2. Bacterial Strains, Gene, and Plasmid

The challenge experiments were conducted using* P. multocida* strains X-73 (serovar A:1, ATCC15742) and P-1059 (serovar A:3, ATCC11039), which as major etiologic strains of fowl cholera are widely considered to be appropriate for assessing the protection of chickens against infection with all* P. multocida* strains. They were grown in brain heart infusion broth (BHI; Merck, Darmstadt, Germany) for 6 h at 37°C. Then the bacteria were subcultured onto a blood agar and incubated at 37°C for 18 h.* E. coli* strain PQE-ompH, which carried the 6×Histidine tag fused* omp*H gene of the* P. multocida* strain X-73 plasmid in the* E. coli* strain M15 from our previous study [3], was grown in Luria-Bertani (LB) broth or on LB agar containing 100 *μ*g/mL ampicillin and 25 *μ*g/mL kanamycin (Sigma-Aldrich, St. Louis, MO, USA) at 37°C.

### 2.3. Purification of Native OmpH

Native OmpH was prepared using the electroelution method as described elsewhere [8]. Briefly, crude capsular extract (CCE) was prepared using the saline extraction method as described previously [8]. Then, target 39 kDa protein of native OmpH was purified by electroelution (electroelution electrophoresis apparatus, ATTO) in 20 mM Tris base, 150 mM glycine, and 0.01% SDS buffer at 100 V for 1 h in an icebox. The conditions for protein collection were 200 min for delay time, 2 min for EP time, 100 s for filling time, 120 s for collecting time, and 15 mA for electrical current. The eluted native OmpH was passed through the detergent removing minicolumn (Ampure DT, Amersham, Japan). Then, the total protein in the supernatant was quantified using the BCA protein assay kit (Pierce, Rockford, IL, USA) and the eluted protein was kept at −20°C until use.

### 2.4. Preparation and Purification of rOmpH

The rOmpH was expressed via* E. coli* strain PQE-ompH as described in our previous study [3]. Then, the expression of the recombinant protein was induced by the addition of isopropyl-*β*-D-thiogalactopyranoside (IPTG; Amresco, Solon, OH, USA) to a final concentration of 1 mM and continually incubated under the same conditions for a further 5 h. Finally, the bacterial cells were harvested by centrifugation at 4,000 ×g at 4°C for 20 min. The supernatant was discarded and the cell pellets were stored in −20°C for further utilization.

*E. coli* cell pellets were lysed and purified using the electroelution method or a denatured condition of affinity chromatography [1]. Purification of the recombinant protein using the electroelution method was explained as follows. The 5 g wet weight of cell pellets was lysed in 10 mL of a native lysis buffer (50 mM NaH_2_PO_4_, 300 mM NaCl, and 10 mM imidazole, pH 8.0). The solution was gently mixed, at 4°C, and then the homogenate was centrifuged at 10,000 ×g at 4°C. The supernatant containing 1,500 *μ*g of total protein was run on a preparative 12.5% sodium dodecyl sulfate polyacrylamide gel column (10 mm stacking gel and 30 mm separating gel) in a sample buffer (4% SDS, 50 mM, Tris, 20% of glycerol, and 0.005% of bromophenol blue) using the electroelution apparatus (Nativen, ATTO, Tokyo, Japan). The conditions for protein collection were 200 min for delay time, 2 min for EP time, 100 s for filling time, 120 s for collecting time, and 15 mA for electrical current. Protein fractions were collected in buffer (371 mM Tris, 5% sucrose, pH 8.8) and the total protein was quantified using the BCA protein assay kit (Pierce) before being kept at −20°C.

### 2.5. Protein Fraction Analyses

Each 10 *μ*g protein fraction was identified on the basis of presence of the target protein by 12.5% SDS-PAGE according to the Laemmli method [12] and then subjected to western blotting. Protein fractions were transferred onto nitrocellulose membrane and immunostained with an anti-HisG-HRP antibody (Invitrogen, Carlsbad, CA, USA). After incubation with antibodies, the membranes were washed thoroughly with PBST. The protein bands were visualized following incubation with 3,3′-diaminobenzidine (DAB; Invitrogen) as a chromogenic substrate.

### 2.6. Determination of Antibody Responses

Specific antibody responses of the chicken sera were determined through measuring the Immunoglobulin Y (IgY) titers using a commercial indirect ELISA test kit for fowl cholera (ProFLOK, Synbiotics, Kansas City, MO, USA). The plates were evaluated using an ELISA plate reader (Immuno Mini NJ 2300, Intermed, Japan) and the average sample per positive (S/P) ratio of each group was calculated according to the manufacturer's recommendation. The S/P ratios were calculated according to the following equation: S/P ratio (%) = [corrected optical density of a sample/corrected optical density of a positive reference serum]. There is no quantitative test sensitivity and specificity information available for this test.

### 2.7. Chicken Embryo Fibroblast (CEF) Cell Culture

The CEF cells were obtained from 10-day-embryonated chicken eggs (The Upper Northern Veterinary Research and Development Center, Hang Chat, Lam Pang, Thailand). Approximately 48 h before the experiment, a total of 2.5 × 10^5^ cells/mL in 2 mL Dulbecco's Modified Eagle Medium (DMEM; Invitrogen) supplemented with 5% fetal bovine serum (Invitrogen), 1% L-glutamine (Invitrogen), and 100 U/mL of penicillin and streptomycin (Invitrogen) were seeded into 35 mm Corning culture dishes containing 22 × 22 mm cover slips in the bottom of the well. The dishes were incubated at 37°C with 5% CO_2_. After the incubation, the dishes were washed three times with 2 mL of sterile PBS pH 7.4 and used for the adhesion inhibition assay.

### 2.8. Adhesion Inhibition Assay

The adhesion inhibition assay was modified based on a previous study [8]. Briefly,* P. multocida* strains were grown separately on blood agar at 37°C for 18 h and were resuspended in sterile PBS and suspension turbidity was adjusted to 0.5 of McFarland Standard (approximately 2.8 × 10^8^ cfu/mL) at the wavelength of 600 nm. The day after vaccination with the highest average S/P value in the indirect ELISA across all serum samples was identified, and samples from that day were pooled within each of the 10 treatment-challenge groups and used for this assay. To represent the bacterial challenge* in vitro*, for each of the 10 treatment-challenge groups 2 mL of the bacterial suspension was added to 3 mL of pooled chicken serum and incubated at 37°C for 1 h. After the incubation, the resulting suspension was inoculated onto the monolayer of CEF cells and incubated at 37°C with 5% CO_2_ for 1 h. Nonadherent bacteria were removed by washing with 2 mL of sterile PBS. The washing step was repeated for 4 times. After washing the cover slips were fixed with 4% formaldehyde and stained with Wright Giemsa solution (Sigma-Aldrich). The cover slips were examined under a light microscope with 1000x power of magnification. For each of the treatment-challenge subgroups, 100 CEF cells with intact structure were selected and the number of adhering bacteria was counted. The selection of CEF cells occurred randomly by scanning a magnification field from the left to the right and from the top to the bottom of the cover slips. The counts for each selected CEF cell were performed twice and the mean was used in the analysis.

### 2.9. Experimental Infection in Chickens

After allowing two weeks following the second immunization for chickens to develop an immune response, chickens in all groups were challenged with the bacteria by intramuscular injection of 1 mL of bacterial suspension containing 2 × 10^6^ cfu/mL of strain X-73 or 4.3 × 10^6^ cfu/mL of P-1059 [3], respectively. The birds were examined for clinical signs over a 7-day postchallenge period, and mortality was recorded.

### 2.10. Statistical Analysis

The results were analyzed using Stata SE 13.1 software (StataCorp LP, College station, TX, USA) and IBM SPSS Statistics version 22. No statistical analysis was performed for the antibody response data as only summary statistics were available. A general linear model analysis was used to compare the average adhesion inhibition of bacteria to CEF cells counts between treatment groups and* in vitro* infection challenge groups, as well as their interaction. The means between different groups were compared using simple contrasts. The survival of chickens was compared between different treatment and* in vivo* infection challenge groups using binary logistic regression. Exact estimation was used given the small sample sizes in this study.

## 3. Results

### 3.1. Expression and Purification of rOmpH

**Figure 1:**
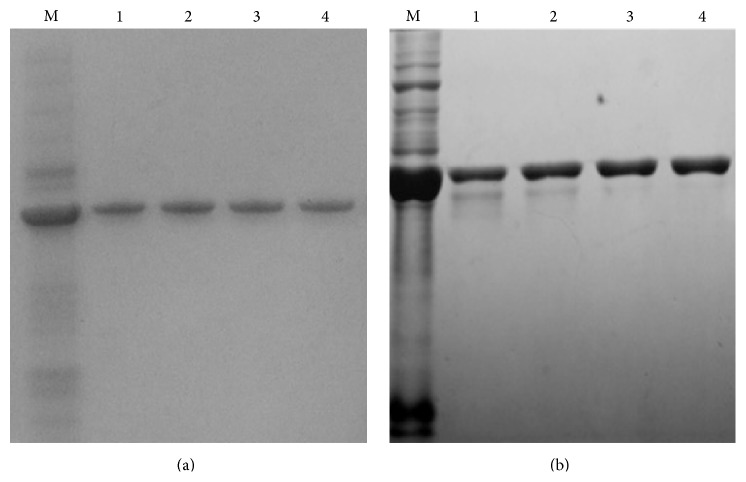
The fractions of rOmpH purified by electroelution were analyzed using SDS-PAGE (a) and probed with anti-HisG-HRP antibody (b). Lane M: cell lysates of the* E. coli *host. Lanes 1–4: rOmpH fractions purified using electroelution.

**Figure 2:**
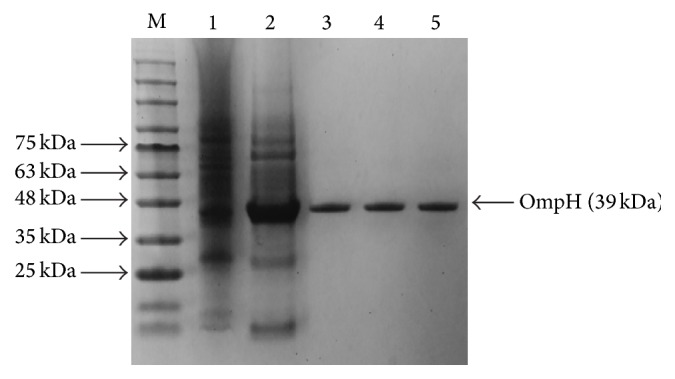
SDS-PAGE of proteins used in this study. Lanes: M: molecular mass standards; 1: whole cell lysate of strain X-73; 2: prepurified rOmpH; 3: rOmpH purified by electroelution; 4: rOmpH purified by a hybrid condition of affinity chromatography; and 5: native OmpH of strain X-73 purified by electroelution.

*E. coli* cell whole cell lysates showed an overexpressed band at approximately 39 kDa on sodium dodecyl sulfate polyacrylamide gel electrophoresis (SDS-PAGE; Figure 1). The rOmpH fractions from the electroelution apparatus, which was employed in order to check whether the eluted fractions had been extracted, showed a single protein band with the same target molecular mass, as shown in Figure 1. The western blotting, probed with anti-6×Histidine tagged-antibody, also confirmed the overexpressed band of rOmpH which was tagged with the 6×Histidine, as shown in Figure 1. Additionally, all the proteins used in this study were analyzed using SDS-PAGE (Figure 2). The molecular mass of those 3 proteins, native OmpH, and the two types of rOmpH produced using different purification methods were identical with approximately 39 kDa.

### 3.2. Antibody Responses after Vaccination

**Figure 3:**
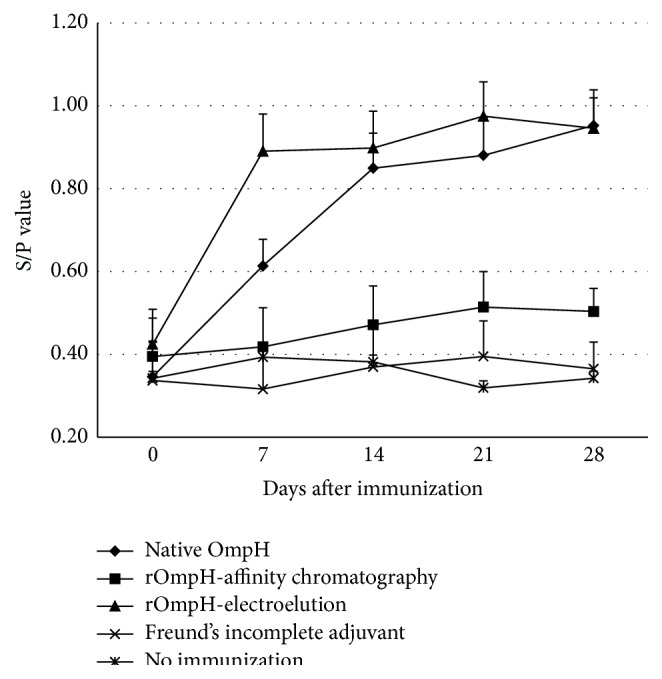
The temporal pattern of the average S/P value of chicken sera (including standard error) based on indirect ELISA (ProFLOK) for each of the five treatment groups (first vaccination on day 0 and the second on day 14).

It is to be noted that the findings in relation to antibody data are based on visual analysis of the data, since only summary statistics were available. The levels of serum antibody in the chickens immunized with native OmpH or rOmpH, both purified using the electroelution method, increased following the first and second immunizations (Figure 3). Immune responses to native OmpH reached a maximal point 7 days after first dose and began to plateau after 14 days but responses were also slightly increased at days 21 and 28. In contrast, rOmpH reached a maximal point after 7 days and began to plateau at day 7 after first dose. Moreover, responses were also slightly increased at day 21 (rOmpH) but do not appear to be significantly different to the responses at day 14 and therefore were considered to have reached a maximum at day 14. In contrast, low antibody levels were observed in the chickens immunized with the rOmpH purified using affinity chromatography, those receiving incomplete Freund's adjuvant, and the nonimmunized groups.

### 3.3. Bacterial Adhesion following* In Vitro* Challenge with* P. multocida* Strains

**Figure 4:**
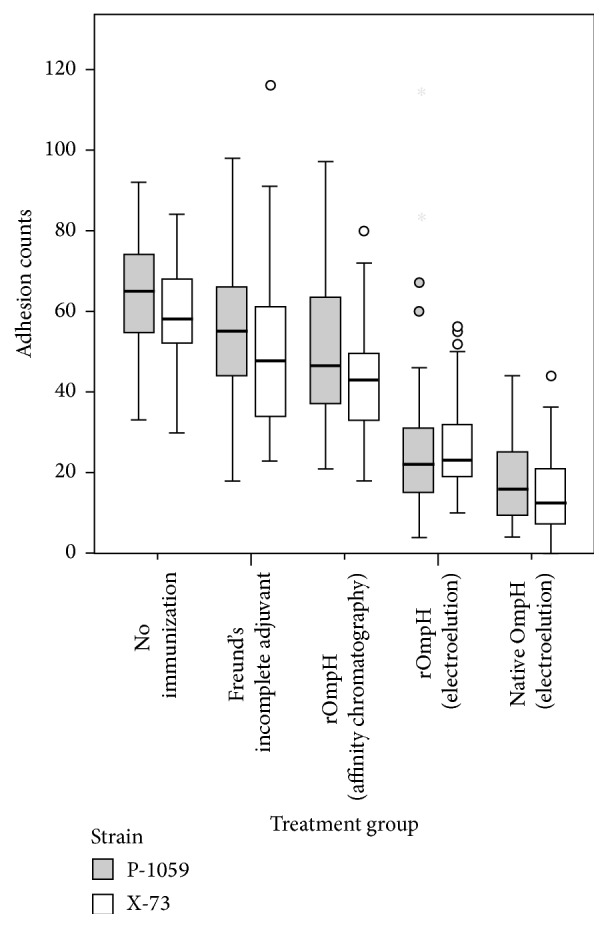
Box-and-whisker plot of counts of adhesions to CEF cells by treatment group and* P. multocida* challenge strains.

The general linear model analysis indicates that the mean number of adherent bacteria differs amongst treatment groups (*p* < 0.001) and between the two challenge strains (*p* < 0.001). There was no statistically significant interaction between treatment group and challenge strain (*p* = 0.07). Compared with the counts for the nonvaccinated group, the samples from chickens immunized with Freund's incomplete adjuvant had average bacterial adhesion counts per CEF which were lower by 8.9 (95% CI 5.9–11.9), those immunized with rOmpH purified using affinity chromatography were lower by 15.0 (95% CI 12–18), those immunized with rOmpH purified using electroelution were lower by 35 (95% CI 32.4–38.5), and those immunized with OmpH purified using electroelution were lower by 45 (95% CI 42.6–48.2). The pooled sera challenged* in vitro* with* P. multocida* strain P-1059 had on average 4.1 (95% CI 2.3–5.9) more adherent bacteria per CEF cell than those challenged with X-73. The distributions of counts of bacteria adherent to CEF cells for each of the 10 treatment-challenge groups are shown in Figure 4.

### 3.4. Survival following* In Vivo* Infection Challenge with* P. multocida* Strains

Amongst chickens developing clinical disease, clinical signs were observed from 12 hours following bacterial challenge. There was no statistically significant difference in survival between the two challenge strain groups, and this effect did not vary between treatment groups (median unbiased estimate of odds ratio = 1, exact 95% CI 0.19–5.1). There was a statistically significant difference in survival between treatment groups. Using nonvaccinated chickens as the reference group, there was a higher survival proportion in chickens immunized with either OmpH (median unbiased estimate of odds ratio = 238, exact 95% CI 33–infinity) or rOmpH purified using electroelution against challenge with one of the two* P. multocida* strains (median unbiased estimate of odds ratio = 55, exact 95% CI 9–infinity). The other two groups did not differ in their survival from the reference group. Table 1 shows the proportions of surviving chickens for each treatment and* P. multocida* strain challenge group.

## 4. Discussion

The structural integrity of the expressed protein is one of the main concerns in recombinant protein production. This is due partly to the original protein structure having an important role in inducing specific antibodies. The antigenic epitope and structure of the recombinant protein must also be conserved during the purification process. Electroelution is used to extract a particular protein of interest from an electrophoresis gel by applying an electric current [6, 13]. The method is considered to be an effective purification method for separating a small target band of proteins from a crude whole protein sample [7–10]. Other chromatography methods are less able to uniquely separate the target protein band [6]. The electroelution method uses polyacrylamide gel electrophoresis (PAGE) which allows effective separation of the target protein band from a crude original protein [6, 13]. Indeed, PAGE is routinely used to determine protein or nucleotide purity. Amongst its advantages is also the absence of sodium dodecyl sulfate (SDS) and urea in the gel composition. In the current study, electroelution was applied to purify the rOmpH from cell lysate in order to protect the immunogenicity of the protein.

Outer membrane protein H (OmpH) is a porin protein which is considered to be highly conserved among Gram-negative bacteria including* P. multocida* strains [14]. A native form of OmpH is a homotrimer of approximately 110 kDa while a monomeric form of this protein can be obtain by induced denaturation and ranges from 34 to 42 kDa. The variation in the size of the monomer depends on the serotype and the electrophoretic system used for the analysis [1, 15, 16]. Chevalier et al. [15] demonstrated the use of size exclusion chromatography to purify the native form of OmpH and successfully protected the immunogenicity of the protein. Subsequently, Luo et al. [1] employed the same method and used the protein as an immunogen in chickens. The native form of OmpH produced effective protection in chickens against homologous challenge-exposure. Recombinant OmpH of* P. multocida* strain X-73 has been cloned and expressed by Luo et al. [1]. The rOmpH was purified by a denatured condition of affinity chromatography and characterized the immunogenicity. The protection in chickens conferred by immunization with rOmpH in that study was low when compared with a native form of OmpH. Luo et al. [1] suggested that the structure of the recombinant outer membrane protein H (rOmpH) of avian* P. multocida* strain X-73 had changed and affected its immunogenicity following purification by a denatured condition of affinity chromatography. According to previous reports on immunization with bacterial porins in animal models, the trimeric or native conformation of porin is considered essential for induction of protective immunity [1, 2]. Until now, there has been only one application of the electroelution method for purification of a native form of OmpH (Borrathybay et al., 2003); however, the application for purification of rOmpH has not been demonstrated yet. Borrathybay et al. [8] attempted to purify a native form of OmpH of* P. multocida* strain P-1059 from crude capsular extract. The purified OmpH provided effective protection in chicken against challenge with different* P. multocida* strains. This suggested that the electroelution method used in that study had no adverse effect on the immunogenicity of OmpH after purification. According to the results from the current study, the protection conferred by immunization with OmpH or rOmpH purified using the electroelution method resulted in a higher level of protection amongst chickens compared with other purification methods. This indicates that the electroelution method can be used for purification of rOmpH without adversely affecting its immunogenicity. Furthermore, given that there was no statistically significant difference in the performance of native OmpH and rOmpH both purified using electroelution, it can be concluded that the recombinant OmpH of* P. multocida* can be used as effectively as the native one.

The ability to induce cross-immunity among* P. multocida* serovars is important in the development of poultry vaccines. Similar to this study, Sthitmatee et al. [3] and Borrathybay et al. [8] also demonstrated that OmpH and rOmpH of* P. multocida* strains are cross-protective immunogens against avian* P. multocida* strains. The method used for purification of the protein influences its immunogenicity, including its cross-protection potential. This is important since a natural fowl cholera outbreak can be caused by strains that are different from the vaccination strain or may involve multiple strains. A previous study suggested that the natural expression of the antigen responsible for cross-protection is limited under the* in vitro* growth conditions during the proliferation process [17].

The current study demonstrates that both native OmpH and rOmpH purified using electroelution induce effective* in vitro* antibody protection that inhibits the adhesion of two common avian* P. multocida* strains to CEF cells. In contrast, the antibodies induced by rOmpH purified by a denatured condition of affinity chromatography resulted in a high number of adhesions of bacteria to CEF cells. This result provides strong evidence that the electroelution was successful at refolding the protein conformation. In accordance with the previous study [8], the native form of OmpH induced an efficient antibody which inhibited the bacterium from adhering to CEF cells. Moreover, low amounts of OmpH in bacterial capsule affected the cross-protectivity [18]. However, the method requires further basic biochemistry coupled with bioinformatics tools to verify the protein structure. The result was confirmed in the* in vivo* challenge experiment where survival in the groups immunized with rOmpH purified using electroelution was 85% compared with 25% in the groups immunized with rOmpH purified using affinity chromatography. These* in vitro* and* in vivo* results demonstrate the potential of rOmpH purified using electroelution for protection of chickens against* P. multocida* infection.

## 5. Conclusion

The rOmpH purified using the electroelution method retains its immunogenicity as demonstrated by being able to induce specific antibodies against avian* P. multocida* strains. It successfully protected against homologous strain challenge as measured by* in vitro* and* in vivo* challenge with two different avian* P. multocida* strains. The rOmpH purified using the affinity chromatography method achieved poor protection in the challenge experiment.

## Figures and Tables

**Table 1:** Experimental design and results of *P. multocida* challenge of immunized and nonimmunized chickens.

Treatment groups	Type of immunogen	*P. multocida* strain challenge subgroup Number of survivors/total (% protection)	
		X-73	P-1059
1	rOmpH purified using electroelution	9/10 (90)^*∗*^	8/10 (80)^*∗*^
2	rOmpH purified using a denatured condition of affinity chromatography	2/10 (20)	3/10 (30)
3	Native OmpH purified using electroelution	10/10 (100)^*∗*^	10/10 (100)^*∗*^
4	Incomplete Freund's adjuvant	0/5 (0)	0/5 (0)
5	No immunization	0/5 (0)	0/5 (0)

^*∗*^Significantly difference (*p* < 0.05).

## References

[1] Luo Y., Glisson J. R., Jackwood M. W. (1997). Cloning and characterization of the major outer membrane protein gene (ompH) of Pasteurella multocida X-73. *Journal of Bacteriology*.

[2] Rimler R. B. (2001). Purification of a cross-protective antigen from *Pasteurella multocida* grown *in vitro* and *in vivo*. *Avian Diseases*.

[3] Sthitmatee N., Numee S., Kawamoto E. (2008). Protection of chickens from fowl cholera by vaccination with recombinant adhesive protein of *Pasteurella multocida*. *Vaccine*.

[4] Chellan B., Appukuttan P. S., Jayakumari N. N. (2008). Electroelution of lipoprotein(a) [Lp(a)] from native polyacrylamide gels: a new, simple method to purify Lp(a). *Journal of Biochemical and Biophysical Methods*.

[5] Sá-Pereira P., Duarte J., Costa-Ferreira M. (2000). Electroelution as a simple and fast protein purification method: isolation of an extracellular xylanase from *Bacillus* sp. CCMI 966. *Enzyme and Microbial Technology*.

[6] Shoji M., Kato M., Shuichi H. (1995). Electrophoretic recovery of proteins from polyacrylamide gel. *Journal of Chromatography A*.

[7] Barrell P. J., Liew O. W., Conner A. J. (2004). Expressing an antibacterial protein in bacteria for raising antibodies. *Protein Expression and Purification*.

[8] Borrathybay E., Sawada T., Kataoka Y. (2003). A 39 kDa protein mediates adhesion of avian *Pasteurella multocida* to chicken embryo fibroblast cells. *Veterinary Microbiology*.

[9] Kaur U., Khurana S., Saikia U. N., Dubey M. L. (2013). Immunogenicity and protective efficacy of heparan sulphate binding proteins of *Entamoeba histolytica* in a guinea pig model of intestinal amoebiasis. *Experimental Parasitology*.

[10] Pinto A. R., Beyrodt C. G. P., Lopes R. A. M., Barbiéri C. L. (2000). Identification of a 30 kDa antigen from *Leishmania* (*L*.) *chagasi* amastigotes implicated in protective cellular reponses in a murine model. *International Journal for Parasitology*.

[11] American Veterinary Medical Association (2013). *AVMA Guidelines for the Euthanasia of Animals*.

[12] Laemmli U. K. (1970). Cleavage of structural proteins during the assembly of the head of bacteriophage T4. *Nature*.

[13] Harrington M. G., Deutscher M. P. (1990). Purification procedures: electrophoretic methods elution of protein from gels. *Guide to Protein Purification, Methods in Enzymology*.

[14] Hatfaludi T., Al-Hasani K., Boyce J. D., Adler B. (2010). Outer membrane proteins of *Pasteurella multocida*. *Veterinary Microbiology*.

[15] Chevalier G., Duclohier H., Thomas D., Shechter E., Wroblewski H. (1993). Purification and characterization of protein H, the major porin of *Pasteurella multocida*. *Journal of Bacteriology*.

[16] Lübke A., Hartmann L., Schröder W., Hellmann E. (1994). Isolation and partial characterization of the major protein of the outer membrane of *Pasteurella haemolytica* and *Pasteurella multocida*. *Zentralblatt für Bakteriologie*.

[17] Rebers P. A., Heddleston K. L. (1977). Fowl cholera: induction of cross-protection in turkeys with bacterins prepared from host-passaged *Pasteurella multocida*. *Avian Diseases*.

[18] Sthitmatee N., Yano T., Lampang K. N., Suphavilai C., Kataoka Y., Sawada T. (2013). A 39-kDa capsular protein is a major cross-protection factor as demonstrated by protection of chickens with a live attenuated *Pasteurella multocida* strain of P-1059. *Journal of Veterinary Medical Science*.

